# Nickel-Based Metal-Organic Frameworks as Electrocatalysts for the Oxygen Evolution Reaction (OER)

**DOI:** 10.3390/molecules27041241

**Published:** 2022-02-12

**Authors:** Linda Sondermann, Wulv Jiang, Meital Shviro, Alex Spieß, Dennis Woschko, Lars Rademacher, Christoph Janiak

**Affiliations:** 1 Institut für Anorganische Chemie und Strukturchemie, Heinrich-Heine-Universität Düsseldorf, 40225 Düsseldorf, Germany; linda.sondermann@hhu.de (L.S.); alex.spiess@hhu.de (A.S.); dennis.woschko@hhu.de (D.W.); lars.rademacher@hhu.de (L.R.); 2 Forschungszentrum Jülich GmbH, Institute of Energy and Climate Research, IEK-14: Electrochemical Process Engineering, 52425 Jülich, Germany; j.wulv@fz-juelich.de

**Keywords:** metal-organic frameworks (MOF), electrocatalysis, oxygen evolution reaction (OER), nickel, ketjenblack

## Abstract

The exploration of earth-abundant electrocatalysts with high performance for the oxygen evolution reaction (OER) is eminently desirable and remains a significant challenge. The composite of the metal-organic framework (MOF) Ni_10_Co-BTC (BTC = 1,3,5-benzenetricarboxylate) and the highly conductive carbon material ketjenblack (KB) could be easily obtained from the MOF synthesis in the presence of KB in a one-step solvothermal reaction. The composite and the pristine MOF perform better than commercially available Ni/NiO nanoparticles under the same conditions for the OER. Activation of the nickel-cobalt clusters from the MOF can be seen under the applied anodic potential, which steadily boosts the OER performance. Ni_10_Co-BTC and Ni_10_Co-BTC/KB are used as sacrificial agents and undergo structural changes during electrochemical measurements, the stabilized materials show good OER performances.

## 1. Introduction

The depletion of fossil fuels and their direct correlation in increasing global greenhouse gas emissions through their combustion show that the development of new sustainable clean energy sources is required [1,2,3]. A possible solution is coupling renewable energy sources like solar and wind energy with electrochemical water splitting to convert surplus electrical energy into storable hydrogen fuel [4,5,6,7]. Electrochemical water splitting consists of two half reactions, the cathodic hydrogen evolution reaction (HER; alkaline conditions: 4 H_2_O + 4 e^−^ → 2 H_2_ + 4 OH^−^, E° = 0.00 V vs. RHE) and the anodic oxygen evolution reaction (OER, alkaline conditions: 4 OH^−^ → O_2_ + 4 e^−^ + 2 H_2_O, E° = 1.23 V vs. RHE) [8,9]. The OER involves a four electron-proton coupled transfer process to generate one oxygen molecule and occurs at an applied potential (overpotential η) much higher than the theoretical equilibrium potential of E° = 1.23 V vs. RHE [8,10,11,12,13,14]. The overpotential is the difference between the applied potential and the equilibrium potential

(1) η_OER_ = E_RHE_ − E° (1.23 V)

and it is one of the key parameters on which the performance of an electrocatalyst is evaluated on [9,15,16]. Ideally a highly active electrocatalyst produces a large current density with a small overpotential [11] together with long-term stability. Another key parameter, which gives insight into the reaction mechanism and rate-determining step, is the Tafel slope (b), which can be obtained through the Tafel-equation [12,17,18].

(2) η=a+b×log(j)

Up to date electrocatalysts based on noble metals, such as ruthenium and iridium, together with their oxides RuO_2_ and IrO_2_, are the OER-catalysts with the best performance, independently of the pH of the electrolyte [19,20,21]. However, the scarcity and high costs of these noble metals severely hinder a large-scale industrial application. Subsequent research has been directed towards the development of non-noble metal alternatives for the OER [22,23]. Research focuses on finding active, stable and inexpensive electrocatalysts, where especially 3d-transition-metals such as Fe, Ni and Co are of high interest [7,24,25]. Recently metal-organic frameworks (MOFs) and MOF-based electrocatalysts have been paid much attention [26,27]. MOFs are potentially porous, crystalline coordination networks with metal nodes and bridging organic ligands [28,29]. MOFs have been investigated as electrocatalysts due to their high porosity, large surface areas, diversity of composition and structure [27,30,31,32,33]. MOFs can be used directly for electrocatalytic reactions, but they have drawbacks like (i) low electrical conductivity, (ii) mass transport problems of reactants, products and electrolyte ions through their micropores, (iii) lack of stability especially in highly acidic or alkaline aqueous environments [34]. Because of those drawbacks, MOFs are often employed as precursors or sacrificial agents to construct structured carbon-based metal oxide materials as efficient electrocatalysts [27,32,35]. To increase the low electric conductivity and the electrocatalytic performance of MOFs carbon supports are added such as graphene [36], carbon nanotubes (CNTs) [21] or ketjenblack (KB) [37]. The performance of Ni-MOF electrocatalysts could be enhanced by the introduction of a second metal such as cobalt or iron [35,38,39,40,41]. Our previous work has shown that a Ni(Fe)-MOF/KB composite exhibited remarkable OER performance [34]. The MOF Ni-BTC and Ni(Fe)-BTC (BTC = 1,3,5-benzenetricarboxylate) have a fast charge transfer rate and high activity for the OER [40,42]. A mixed-metal Ni(Co)-BTC MOF has only been used as a sacrificial agent to give NiCo_2_O_4_ [35], which encouraged us to take a closer look at the activity of the pristine MOF and the composite material including KB for the OER in this work.

## 2. Results and Discussion

### 2.1. Synthesis and Characterization of the Ni-BTC Analogs

**Figure 1:**
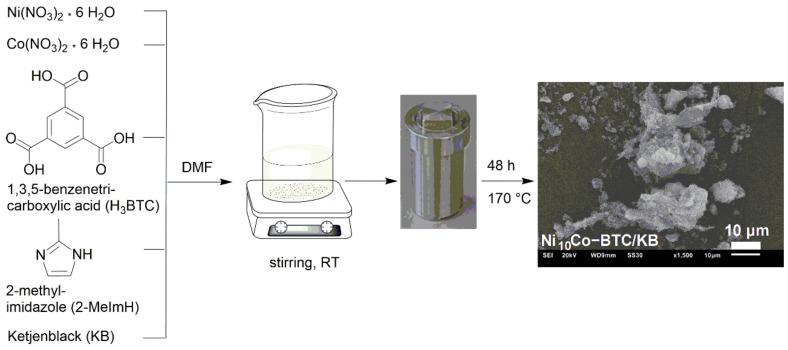
Schematic illustration of the Ni_10_Co-BTC/KB composite synthesis.

The Ni-BTC structure is identical to the Cu-BTC or HKUST-1 structure (HKUST = Hong Kong University of Science and Technology) [43] both are built by dinuclear metal(II)-secondary building units (SBUs), which are connected by BTC in a paddle-wheel fashion to a three-dimensional network of formula [Ni_3_(BTC)_2_] [43,44]. The composite of Ni_10_Co-BTC and KB, named Ni_10_Co-BTC/KB was generated through a facile MOF synthesis in the presence of ketjenblack (KB) in a one-step solvothermal reaction at 170 °C for 48 h from a mixture of Ni(NO_3_)_2·_ 6 H_2_O, Co(NO_3_)_2·_ 6 H_2_O (molar Ni:Co ratio 10:1), 1,3,5-benzenetricarboxylic acid (H_3_BTC), 2-methylimidazole (2-MeImH), and KB in N,N-dimethylformamide (DMF) (Figure 1). Ni_10_Co-BTC and Ni_10_Fe-BTC were synthesized for comparison. Ni-BTC and the mixed-metal analogs are obtained as the dimethylamine adduct [Ni_3_(BTC)_2_(Me_2_NH)_3_] at the axial metal position with Me_2_NH being a hydrolysis product of DMF [43] (from the CHN elemental analysis data in Table S1, Supporting Information (SI)).

A well-known phenomenon in mixed-metal MOF synthesis is that the incorporated metal ratio can differ from the starting material ratio and must be post-synthetically quantified. To quantify the amount of Co and Fe in the synthesized Ni_10_Co-BTC and Ni_10_Fe-BTC flame atomic absorption spectroscopy (AAS) was conducted post-synthetically, resulting in a molar Ni:Co ratio of 11:1 for Ni_10_Co-BTC and a molar Ni:Fe ratio of 11:1 for Ni_10_Fe-BTC (Table 1).

From the AAS determined metal wt.%, the mass fractions of the MOF in the KB composites were calculated as 67 wt.% Ni_10_Co-BTC together with 33 wt.% KB.

**Figure 2:**
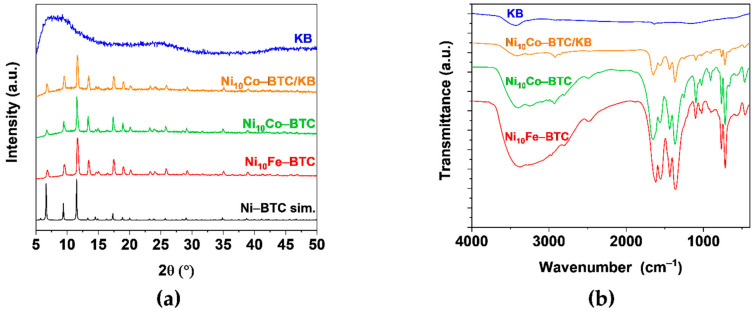
(**a**) PXRD patterns of experimental Ni_10_Fe-BTC (red), Ni_10_Co-BTC (green), Ni_10_Co-BTC/KB (orange), KB (blue) and simulated Ni-BTC (black) (CCDC Nr. 802889); (**b**) FT-IR spectra of Ni_10_Fe-BTC (red), Ni_10_Co-BTC (green), Ni_10_Co-BTC/KB (orange) and KB (blue).

Powder X-ray diffraction (PXRD) patterns of Ni_10_Fe-BTC, Ni_10_Co-BTC, Ni_10_Co-BTC/KB, KB and a simulated diffraction pattern of Ni-BTC are illustrated in Figure 2a. The pristine MOF samples Ni_10_Fe-BTC and Ni_10_Co-BTC exhibit the same reflexes as the simulated pattern of Ni-BTC. The composite Ni_10_Co-BTC/KB also demonstrated a PXRD pattern, which agrees to the simulated pattern of Ni-BTC and also the pristine MOF Ni_10_Co-BTC. The aforementioned PXRD patterns show that the addition of amorphous KB in the synthesis did not influence the MOF crystal growth and structure significantly [45,46]. Apart from the reflexes of the simulated Ni-BTC MOF there are no additional reflexes in the diffraction patterns of the samples, which reveals that no iron or cobalt(oxy)hydroxides formed in the synthesis. This indicates that the second metal (iron or cobalt) was well incorporated into the structure of the Ni-BTC MOF. Pure KB displayed three broad diffraction peaks corresponding to the (100), (002), (101) planes of amorphous carbon [47,48].

Fourier transform infrared (FT-IR) spectra (Figure 2b) of the Ni-BTC analogs demonstrate the same characteristic bands (listed in Table S2, SI) which are in a good agreement with the literature [39]. A broad band in the region of 3600–3000 cm^–1^ and a band around 1650 cm^−1^ can be attributed to the stretching and bending vibrations of -OH group from adsorbed or coordinated water [43,49,50,51]. Characteristic vibrations of a Ni-O bond 577–460 cm^−1^ [51] and of carboxylate-groups 1617–1556 cm^−1^ (asymmetric vibration) and 1439–1364 cm^−1^ (symmetric vibration) can be observed in all samples [50,52]. Vibrations of a Fe-O bond are reported at 538 and 634 cm^−1^ and of a Fe_2_Ni-O bond at ca. 720 cm^−1^, which is also in the range of the vibration of a Co-O bond (725 cm^−1^) [53,54,55]. Fe-O could not be detected and the Fe_2_Ni-O and Co-O bonds are all in a similar range to each other and to aromatic vibrations (Table S2, SI).

**Figure 3:**
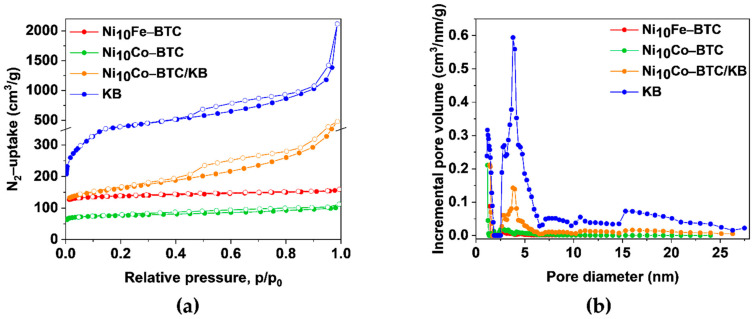
(**a**) N_2_-sorption isotherms at 77 K (Adsorption: filled circles; desorption: empty circles), (**b**) pore size distributions of Ni_10_Fe-BTC (red), Ni_10_Co-BTC (green), Ni_10_Co-BTC/KB (orange) and KB (blue).

The specific Brunauer-Emmett-Teller (BET) surface areas and pore volumes of the materials were derived from nitrogen-adsorption isotherms at 77 K (Figure 3a) and are given in Table S3, SI.

The BET surface areas of the Ni-BTC analogs (Ni_10_Fe-BTC: 555 m^2^/g, Ni_10_Co-BTC: 303 m^2^/g and Ni_10_Co-BTC/KB: 596 m^2^/g) are all in the range of reported values for neat Ni-BTC (Ni-BTC_DMF/EtOH_: 198 m^2^/g and 252 m^2^/g, Ni-BTC_EtOH_: 551 m^2^/g; Ni-BTC: 0.286 cm^3^/g) [56,57]. Ni_10_Fe-BTC and Ni_10_Co-BTC show a type I isotherm revealing their microporosity with a steep gas uptake at low relative pressure followed by a plateau [58,59,60]. KB is a porous carbon material with a BET surface area of 1415 m^2^/g, a pore volume of 1.59 cm^3^/g (Table S3, SI) and mesopores, which are mostly around 4 ± 2 nm (Figure 3b). The adsorption isotherm branch of KB is a composite of a type I and II isotherm and the desorption isotherm branch additionally displays a H4 hysteresis, both being often indicators for micro-mesoporous carbons [58]. The nitrogen sorption isotherm and BET-surface area of the composite Ni_10_Co-BTC/KB is a superposition of the isotherms of the MOF and KB components (Figure 3a). This superposition also holds for the pore-size distribution of the individual components in the composite (Figure 3b). The bimodal pore size distribution of Ni_10_Co-BTC/KB with maxima at ~2 nm and ~4 nm reflects the contributions from the MOF and KB. Consequently, the BET surface area and pore volume of the Ni_10_Co-BTC/KB composite with 596 m^2^/g and 0.45 cm^3^/g, respectively, are higher than the surface area and pore volume of neat Ni_10_Co-BTC (303 m^2^/g, 0.15 cm^3^/g) but still lower than the calculated BET surface area (670 m^2^/g) as determined from the sum of the mass-weighted S(BET) of KB (33 wt.%) and MOF (67 wt.%) (Equation (3) [34]):

(3) $$S(\mathrm{BET}{)}_{\mathrm{calc.}}=\frac{\mathrm{wt}.\%\;\mathrm{of}\;\mathrm{KB}}{100}\;\times \;S(\mathrm{BET}{)}_{\mathrm{KB}}+\frac{\mathrm{wt}.\%\;\mathrm{of}\;\mathrm{MOF}}{100}\times S(\mathrm{BET}{)}_{\mathrm{MOF}}$$

The slightly lower BET surface area can be due to pore blocking effects or formation of the MOF in the mesopores of KB, as evidenced by the large reduction of the incremental mesopore volume in Figure 3b.

Thermogravimetric analyses (TGA) under N_2_ atmosphere yield a similar curvature for all Ni-BTC analogs (Figure S1, SI). In the range of 30–320 °C the initial weight losses until decomposition can be attributed to the loss of crystal solvent molecules (DMF, H_2_O) incorporated in the cavities [61]. After complete solvent loss the BTC-linker together with the MOF structure decomposes around 350–600 °C (Lit. 337–450 °C) (mass change of 54–57 %, Figure S1, SI) [56,60]. The TGA curves are in agreement with reported curves for NiCo-BTC and Ni-BTC [35,43,60,61].

**Figure 4:**
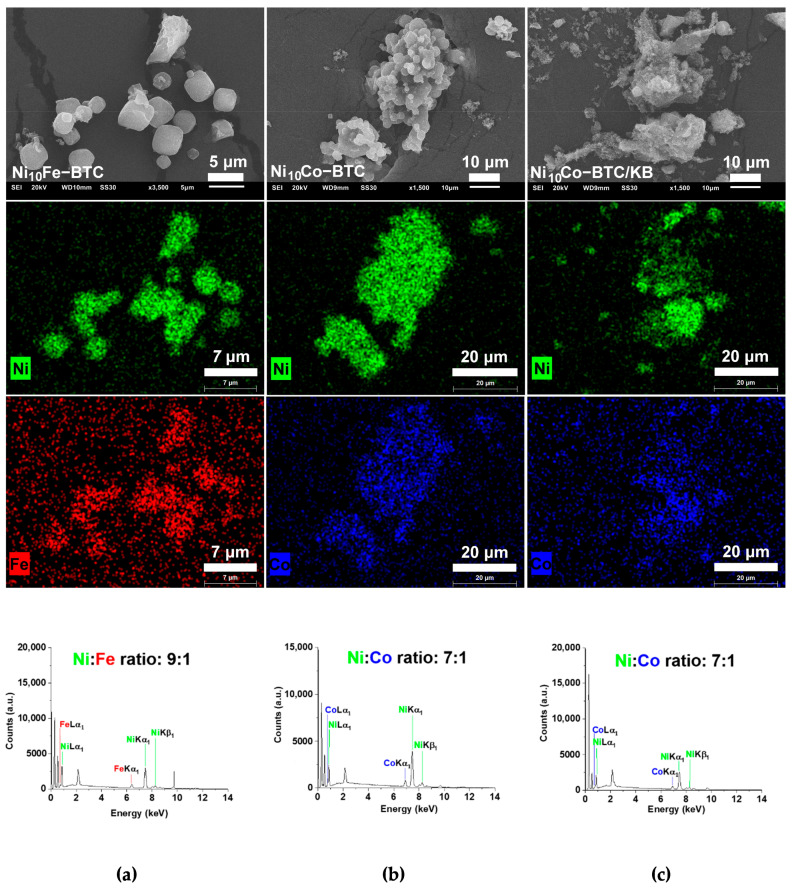
SEM images (**top row**), SEM-EDX metal element mappings (**two middle rows**) and EDX spectra (**bottom row**) for (**a**) Ni_10_Fe-BTC, (**b**) Ni_10_Co-BTC and (**c**) Ni_10_Co-BTC/KB.

Scanning electron microscopy (SEM) images of Ni_10_Fe-BTC (Figure 4a) present spherical and cubic particles, which is in accordance with the literature [40]. Ni_10_Co-BTC (Figure 4b) has irregular shaped aggregates similar to reported Ni-BTC [40]. The KB particles (Figure S3, SI) are smaller than the MOF particles, and do not have a clearly defined shape. In the composite material Ni_10_Co-BTC/KB (Figure 4c) the MOF particles are covered by KB. SEM-energy-dispersive X-ray spectroscopy (EDX) was conducted for the mixed-metal MOFs (Figure 4 and Figure S2, SI) and KB (Figure S3, SI). For the mixed-metal MOFs SEM-EDX metal element mapping (Figure 4) reveals a good superposition of the two different metals. It is evident that the mapping of nickel and iron or cobalt of Ni_10_Fe-BTC and Ni_10_Co-BTC, respectively, is more visible than for Ni_10_Co-BTC/KB, where the KB partially covers and masks surface of the MOF particles.

The metal contents of the mixed-metal samples were quantified by SEM-EDX and atomic absorption spectroscopy (AAS) and are compared in Table 1. The SEM-EDX results are more indicative of the metal ratio of the surface of the samples and the AAS results quantify the metal ratio of the bulk samples. For the synthesis of all materials a starting molar ratio of 10:1 was used for nickel to iron or cobalt. Ni_10_Fe-BTC and Ni_10_Co-BTC give similar AAS results with a ratio of approximately 11:1 (Ni:Fe/Co), which is close to the used 10:1 molar ratio for the synthesis. The AAS of the composite material Ni_10_Co-BTC/KB results in a Ni:Co ratio of approximately 8:1, which is a little lower than the implemented molar ratio in the beginning.

### 2.2. Electrocatalytical Results

The OER performance of all samples was checked using a three-electrode system with rotation disk electrode in 1 mol/L KOH electrolyte. The electrochemical kinetics of the samples were evaluated by comparison of the Tafel slopes derived from linear sweep voltammetry (LSV) curves after the activation. Apart from the overpotential and Tafel slope the stability of the electrocatalysts were examined by comparing the overpotential before and after 1000 cyclic voltammetry cycles (CVs). Details are given in the experimental section.

**Figure 5:**
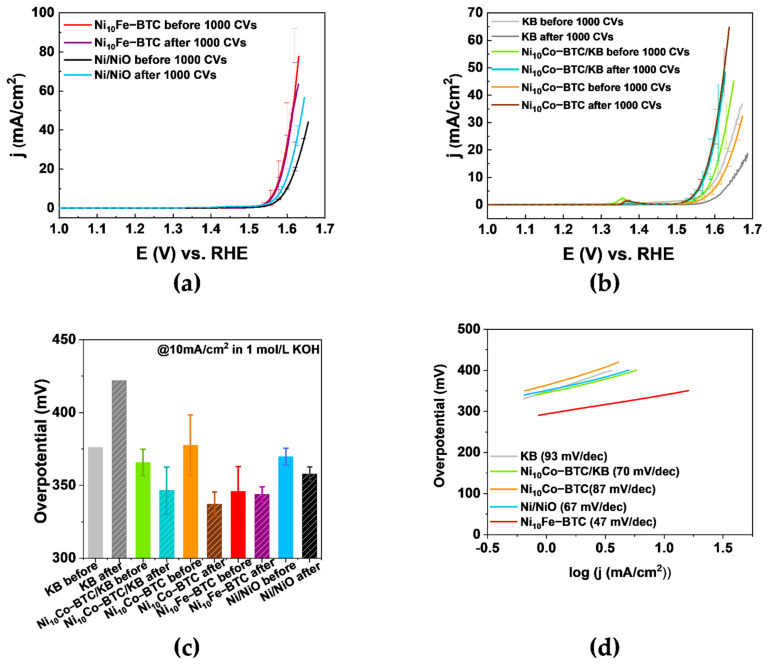
(**a**,**b**) LSV curves of Ni_10_Fe-BTC, Ni/NiO nanoparticles, KB, Ni_10_Co-BTC/KB and Ni_10_Co-BTC before and after 1000 CVs, (**c**) overpotentials calculated from (**a**,**b**), (**d**) Tafel plots.

The LSV curves in Figure 5a,b show that Ni_10_Fe-BTC reaches the highest current density before the stability test in comparison to the reference commercial Ni/NiO nanoparticles, Ni_10_Co-BTC, Ni_10_Co-BTC/KB and KB. The LSV curves in Figure 5b display that the current density of Ni_10_Co-BTC/KB was higher than that of the pristine MOF or KB before the stability test, which could be due to a conductivity enhancement effect by the introduction of KB. After the stability test the achieved current density of the pristine MOF is higher than the composite and both are higher than the current density of KB. The current densities of Ni_10_Fe-BTC and of KB have declined after the stability test. Ni_10_Co-BTC, Ni_10_Co-BTC/KB and the commercial Ni/NiO nanoparticles reach a higher current density after the stability test. The peaks from 1.3 to 1.4 V vs. RHE in Figure 5b originated from the redox reaction of Ni^2+/3+^ [35,62,63]. The redox peaks are less visible for Ni_10_Fe-BTC, which is due to the well-known suppressor effect of Fe for the Ni^2+/3+^ oxidation [64,65,66]. The changed current densities before and after the 1000 CVs already depict that an activation is taking place in case of the NiCo samples and the Ni/NiO nanoparticles. The efficiency of an electrocatalyst is normally checked with the overpotential at a current density (j) of 10 mA/cm^2^, which relates to the approximate current density expected for a 10% efficient solar-to-fuel conversion device under sun illumination [13,67,68]. To have a more defined indicator the initial overpotential and the overpotential after the stability test to reach 10 mA/cm^2^ should be considered. Ni_10_Fe-BTC reaches 10 mA/cm^2^ with an initial overpotential of 346 mV and an overpotential of 344 mV after the stability test. The measurement done after the stability test shows nearly identical values, which indicates that the material is stable in its OER performance. The results show that Ni_10_Fe-BTC has a relatively good OER performance. The overpotentials needed to reach 10 mA/cm^2^ before and after the stability test and Tafel slopes for KB, Ni_10_Co-BTC/KB, Ni_10_Co-BTC and Ni/NiO nanoparticles including the results of Ni_10_Fe-BTC are listed in Table S4, SI. Ni_10_Co-BTC (η = 378 mV → 337 mV), Ni_10_Co-BTC/KB (η = 366 mV → 347 mV) MOF samples and the Ni/NiO nanoparticles (η = 370 mV → 358 mV) all give a decreasing overpotential, which indicates an activation of the materials and an increased OER activity. The improvement of the activity of the materials reveal that the prior activation (10 CVs) was not sufficient and it also can correlate with the formation of, for example, a highly OER active NiOOH layer [69]. Only KB demonstrated a higher overpotential afterwards (η = 376 mV → 422 mV). KB exhibits worse OER activity after 1000 cycles due to carbon corrosion at high potentials in alkaline conditions [70,71]. The carbon corrosion can also be the limiting factor of the composite, since after the 1000 CVs the Ni_10_Co-BTC-derived material provides the lowest overpotential with 337 mV. The Ni_10_Co-BTC/KB composite shows the best initial OER activity, but Ni_10_Co-BTC has a stronger activation after the stability test and consequently a higher activity.

The Tafel slopes (Figure 5d) of Ni_10_Fe-BTC (47 mV/dec), Ni_10_Co-BTC/KB (70 mV/dec), Ni_10_Co-BTC (87 mV/dec) and Ni/NiO nanoparticles (67 mV/dec) are in agreement with the reported values of Ni-BTC (71 mV/dec), FeNi_10_-BTC (60 mV/dec), Fe_3_Ni-BTC (49 mV/dec), FeNi-BTC (50 mV/dec) NiCo_2_O_4_ (Precursor: NiCo-BTC) (59.3 mV/dec) and Ni(OH)_2_ (65 mV/dec) [17,35,40]. The value of the Tafel slope can give insight into the rate determining step (rds) of the OER mechanism. Krasil’shchikov’s OER mechanism is one of the more widely known mechanisms, which is described by Equations (4)–(7) with the corresponding Tafel slopes b [72,73].

(4) $${M+\mathrm{OH}}^{–}\;⇄{\;\mathrm{MOH}+e}^{–}\;\;\;\;b=120\;\mathrm{mV}/\mathrm{dec}$$

(5) $${\mathrm{MOH}+\mathrm{OH}}^{–}⇄{\;\mathrm{MO}}^{–}{+e}^{–}\;\;\;b=60\;\mathrm{mV}/\mathrm{dec}$$

(6) $${\;\mathrm{MO}}^{–}\;\to {\;\mathrm{MO}+e}^{–}\;\;\;\;\;b=45\;\mathrm{mV}/\mathrm{dec}$$

(7) $$2\mathrm{MO}\;\to {\;2M+O}_{2}\;\;\;\;\;b=19\;\mathrm{mV}/\mathrm{dec}$$

For Ni_10_Fe-BTC (47 mV/dec) Equation (6) appears to be the rds of the OER mechanism. The most likely rds of the OER mechanism of Ni_10_Co-BTC/KB (70 mV/dec) and Ni/NiO nanoparticles (67 mV/dec) seem to be Equation (5). The Tafel slope of Ni_10_Co-BTC (87 mV/dec) is in between the values of Equations (4) and (5), which makes it difficult to clearly assign it to one of the two reaction steps.

MOFs often act as sacrificial agents to generate structured carbon-based metal oxide materials as efficient electrocatalysts [27,32,35]. To test the stability of the synthesized MOF materials in the alkaline electrolyte Ni_10_Co-BTC, Ni_10_Co-BTC/KB and Ni_10_Fe-BTC were soaked in 1 mol/L KOH for 24 h.

**Figure 6:**
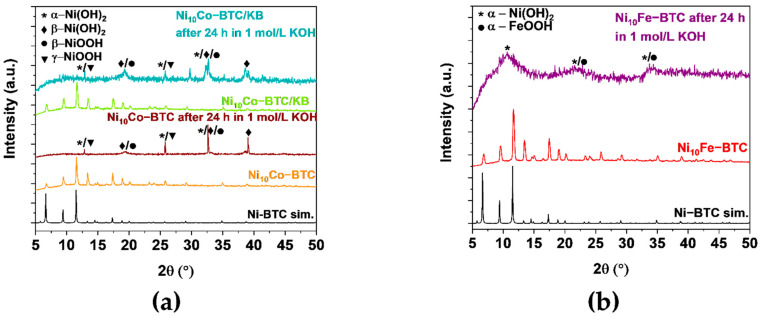
PXRD patterns of (**a**) experimental Ni_10_Co-BTC (orange), Ni_10_Co-BTC/KB (green), Ni_10_Co-BTC, Ni_10_Co-BTC/KB after 24 h in 1 mol/L KOH (brown and dark green) and simulated Ni-BTC (black) (CCDC Nr. 802889), (**b**) experimental Ni_10_Fe-BTC (red), Ni_10_Fe-BTC after 24 h in 1 mol/L KOH (purple) and simulated Ni-BTC (black) (CCDC Nr. 802889); α-Ni(OH)_2_ marked by an asterisk (*) (ICDD: 38-0715), β-Ni(OH)_2_ marked by a diamond (♦) (ICDD:14-0117), β-NiOOH marked by a circle (●) (ICDD:06-0141) and γ-NiOOH marked by a triangle (▼) (ICDD:06-0075) and α-FeOOH marked by a circle (●) (ICDD: 29-0713).

The PXRD patterns of all three samples (Figure 6) display transitions of the MOF structures to their (oxy)hydroxides. Ni_10_Co-BTC and Ni_10_Co-BTC/KB (Figure 6a) exhibit structural changes to α-Ni(OH)_2_ (ICDD:38-0715), β-Ni(OH)_2_ (ICDD:14-0117), β-NiOOH (ICDD:06-0141) and/or γ-NiOOH (ICDD:06-0075) [74]. The relationship between these nickelhydroxides and oxidehydroxides is explained in the Supplementary Information. It is presently not possible, however, to quantify the components in a mixed α/β-Ni(OH)_2_ sample from XPS results [75]. According to literature [75] α- and β-Ni(OH)_2_ could be possibly distinguished from each other by FT-IR spectroscopy. FT-IR spectra for the samples after letting them soak in 1 mol/L KOH for 24 h only indicated also the formation of α- and β-Ni(OH)_2_, albeit without being able to differentiate between them (Figure S7 and Table S5 in the SI). The diffraction patterns for α-Co(OH)_2_ and γ-CoOOH match the given Ni(OH)_2_ and NiOOH diffraction patterns [76]. Similar to Ni_10_Co-BTC and Ni_10_Co-BTC/KB the PXRD pattern of Ni_10_Fe-BTC after 24 h in 1 mol/L KOH (Figure 6b) shows a clear loss of crystallinity of the material and indicates formation of α-Ni(OH)_2_ (ICDD:38-0715) and/or α-FeOOH (ICDD: 29-0713) [74,77]. The change in the structure of Ni_10_Co/Fe-BTC to Ni(OH)_2_, Co(OH)_2_ and/or to NiOOH, CoOOH and/or FeOOH is in agreement to reported observations [78]. The transition to their (oxy)hydroxides fits to the activation which could have been seen through the decrease of their overpotentials (Table S4, SI). Furthermore, transmission electron microscopy (TEM) images were made of the synthesized MOF samples before and after the electrochemical stability tests (1000 CVs). The TEM images (Figures S4–S6, SI) also indicate that a transition of the original MOF morphology takes place. The Ni_10_Co-BTC/KB TEM images (Figure S6, SI) illustrate that the larger MOF particle transformed into nanoparticles. The homogenous Ni_10_Co-BTC MOF particle (Figure S5, SI) changed into a carbon-based material, which contains metal (oxy)hydroxides nanoparticles. The lattice spacings of both NiCo samples (Figures S5d and S6c) could be obtained. The values of the lattice spacings fit to values of reported Ni(OH)_2_ [79] and Co(OH)_2_, which was formed during electrochemical tests of the MOF ZIF-67 [80]. For Ni_10_Fe-BTC (Figure S4, SI) a loss of the former cubic shape of the particle can be observed and out of the resulting new morphology no lattice spacings could be gained. The changed morphology of all samples corroborates the structural changes, which could be seen through the stability test of the synthesized materials in the alkaline electrolyte.

## 3. Materials and Methods

### 3.1. Materials

The used chemicals were obtained from commercial sources and no further purification was carried out. Ketjenblack EC 600 JD was purchased from AkzoNobel, Amsterdam. The Netherlands.

### 3.2. Synthesis of the Ni-BTC Analogs

**Synthesis of Ni_10_Fe-BTC**: Ni_10_Fe-BTC was synthesized according to the literature [40]. 48 mg (0.11 mmol) Fe(NO_3_)_3*_ 9 H_2_O, 349 mg (1.2 mmol) Ni(NO_3_)_2*_ 6 H_2_O, 205 mg (0.98 mmol) H_3_BTC and 55 mg (0.67 mmol) 2-MeImH were dissolved in 15 mL DMF at room temperature (RT) and stirred for 30 min. The prepared solution was transferred into a Teflon-lined stainless-steel autoclave and then heated to 170 °C for 48 h. The resulting dark olive-green precipitate was centrifuged (25 min, 5000 rpm). The precipitate was washed one time with DMF and two times with EtOH and centrifuged again (15 min, 6000 rpm). The product was dried overnight in a vacuum drying cabinet at 90 °C and <50 mbar.

Yield (Ni_10_Fe-BTC): 276 mg

**Synthesis of Ni_10_Co-BTC and Ni_10_Co-BTC/KB:** Ni_10_Co-BTC and Ni_10_Co-BTC/KB were synthesized according to the literature with some modifications [40]. Varying from this synthesis procedure Co(NO_3_)_2*_ 6 H_2_O was used instead of Fe(NO_3_)_3*_ 9 H_2_O for the synthesis of Ni_10_Co-BTC and Ni_10_Co-BTC/KB.

For the Ni_10_Co-BTC sample 35 mg (0.12 mmol) Co(NO_3_)_2*_ 6 H_2_O, 349 mg (1.2 mmol), Ni(NO_3_)_2*_ 6 H_2_O, 205 mg (0.98 mmol) H_3_BTC and 55 mg (0.67 mmol) 2-MeImH were dissolved in 20 mL DMF at RT and stirred for 30 min. For the Ni_10_Co-BTC/KB sample the same amounts were used and 70 mg KB additionally added. The prepared solution was transferred into a Teflon-lined stainless-steel autoclave and then heated to 170 °C for 48 h. The resulting dark olive-green (Ni_10_Co-BTC) and black (Ni_10_Co-BTC/KB) precipitates were centrifuged (25 min, 5000 rpm). The precipitates were washed for one time with DMF and for two times with EtOH and centrifuged again (15 min, 6000 rpm). The products were dried overnight in a vacuum drying cabinet at 90 °C and <50 mbar.

Yield (Ni_10_Co-BTC): 328 mg

Yield (Ni_10_Co-BTC/KB): 357 mg

### 3.3. Materials Characterization

Powder X-ray diffraction (PXRD) measurements were performed at ambient temperature on a Bruker D2 Phaser powder diffractometer with a power of 300 W and an acceleration voltage of 30 kV at 10 mA using Cu-Kα radiation (λ = 1.5418 Å). The diffractograms were obtained on a low background flat silicon sample holder and evaluated with the Match 3.11 software. The samples were measured in the range from 5 to 50° 2θ with a scan speed of 2 s/step and 0.057° (2θ) step size.

Fourier transform infrared spectroscopy (FT-IR) spectra were recorded in KBr mode on a Bruker TENSOR 37 IR spectrometer in the range of 4000–400 cm^−1^.

Nitrogen sorption measurements were performed with a Nova 4000e from Quantachrome at 77 K. The sorption isotherms were evaluated with the NovaWin 11.03 software. Prior to the measurement the materials were first degassed under vacuum (<10^−2^ mbar) at 120 °C for 5 h. Brunauer–Emmett–Teller (BET) surface areas were determined from the N_2_-sorption adsorption isotherms and the pore size distributions were derived by non-local density functional theory (NLDFT) calculations based on N_2_ at 77 K on carbon with slit/cylindrical pores.

Thermogravimetric analyses (TGA) were carried out with a Netzsch TG 209 F3 Tarsus device equipped with an Al crucible with a heating rate of 5 K/min under nitrogen atmosphere.

CHN elemental analyses were conducted with a Vario Mirco Cube from Elementar Analysentechnik.

Flame atomic absorption spectroscopy (AAS) was conducted with a PinAAcle 900T from PerkinElmer. Weighted samples were heated to dryness with 15 mL of aqua regia for two times and afterwards stirred with 10 mL of aqua regia overnight. The solution was carefully filtered and diluted with Millipore water to a volume of 25 mL. The resulting solutions were further diluted with Millipore water (1:50) for the AAS measurements.

Scanning electron microscopy (SEM) images were collected with a JEOL JSM-6510 LV QSEM advanced electron microscope with a LaB_6_ cathode at 20 kV. The microscope was equipped with a Bruker Xflash 410 silicon drift detector and the Bruker ESPRIT software for energy-dispersive X-ray (EDX) analysis which was used to record EDX spectra and EDX mapping. The small amount of Cu, Zn and Au found in the EDX spectra are due to the brass sample holder and the sputtering of the sample with gold prior to the investigation.

Transmission electron microscopy (TEM) images of the MOF samples before the electrochemical tests were recorded on a FEI Tecnai G2 F20 electron microscope operated at 200 kV accelerating voltage equipped with a Gatan UltraScan 1000P detector. TEM samples were prepared by drop-casting the diluted material on 200 μm carbon-coated copper grids. TEM images of the samples after the electrochemical tests were obtained using a FEI Titan, 80–300 TEM with a C_s_ corrector for the objective lens (CEOS GmbH) operated at 300 kV. After the electrochemical test the electrode was rinsed in isopropanol and sonicated until all the layers from the surface of the electrode were dissolved into the solution. Again, the TEM samples were prepared by drop-casting the solution onto the TEM grid. Particle sizes and size distribution were determined manually using the Gatan Digital Micrograph software. For the size distribution over 150 individual particles were analyzed.

### 3.4. Electrocatalytic Measurements

The electrocatalytic OER measurements were conducted with a SP-150 Potentiostat form BioLogic Science Instruments and with a three-electrode setup. As reference electrode a mercury/mercury oxide (Hg/HgO) electrode was used. As counter electrode a Pt wire was used. As working electrode, a rotating disc electrode (RDE), here a glassy carbon electrode (GCE), was used. For the electrocatalyst inks 8 mg of electrocatalyst was dispersed in 1.5 mL isopropanol, 0.5 mL deionized water, and 20 μL Nafion (5 wt.%) and sonicated for 40 min. Catalyst loading was 0.2 mg/cm^2^ by drop casting 10 μL ink on the GC surface (geometric area of 0.196 cm^2^). All the powders dispersed well, forming a stable and homogeneous ink. After drying, the film fully covered the GC electrode. All the electrochemical measurements were conducted in 1 mol/L concentrated Ar-saturated KOH electrolyte, which has been purged with O_2_ for 20 min prior to the OER experiments, with a rotation speed of 1600 rpm at RT. An activation protocol was used before the LSV measurements by cycling the working electrode between 1.0 V and 1.7 V vs. RHE at a scan rate of 100 mV/s for 10 cycles. The LSV polarization curves were recorded in a potential range of 1.0 to 1.7 V vs. RHE at a sweep rate of 5 mV/s without iR correction. The potential applied to the ohmic resistance was extracted later manually. The cycling stability was measured by comparing LSV curves before and after 1000 cycles between 1.0–1.7 V with a scan rate of 100 mV/s.

The measured potentials (vs. Hg/HgO) were converted in potentials vs. RHE with the following Equation (8) [81]:

E_RHE_ = E_(Hg/HgO)_ + 0.059pH + E°_(Hg/HgO)_ (8)

with E_RHE_ = converted potential vs. RHE, E_(Hg/HgO)_ = measured potential and E°_(Hg/HgO)_ = standard potential of the Hg/HgO reference electrode.

The overpotential was calculated as shown in Equation (1): η_OER_ = E_RHE_ − E° (1.23 V). To reduce the experimental contingency error, at least three repeated measurements were carried out for a sample and the average curves with their error bars were compared in the figures. The OER performance of MOF samples were compared with commercial Ni/NiO nanoparticles (Alfa Aesar, Heysham, UK; VWR, International GmbH, Darmstadt, Germany) and KB (AkzoNobel, Amsterdam, The Netherlands).

## 4. Conclusions

Different mixed-metal Ni-BTC analogs with cobalt and iron doping were synthesized, characterized, tested for their performance in the OER and compared to the reference of Ni/NiO nanoparticles and KB. The pristine MOFs Ni_10_Co-BTC and Ni_10_Fe-BTC, as well as the composite Ni_10_Co-BTC/KB could be prepared easily through a one-step solvothermal reaction. To compensate the shortcoming of low MOF conductivity for electrocatalysis, the highly porous and conductive carbon material KB was added, which can also support the transport of electrolyte ions and evolved gases. The MOF electrocatalysts are not stable under the implemented alkaline conditions for the electrocatalytic measurements, which again emphasizes that MOFs can be regarded as sacrificial agents. Nevertheless, the resulting, stabilized materials all evince good performances in the OER. Comparing the overpotentials of Ni_10_Co-BTC (η = 378 mV) and Ni_10_Co-BTC/KB (η = 366 mV) before the stability test, the composite shows a better performance for the OER, but afterward, the Ni_10_Co-BTC-derived electrocatalyst exhibits a lower overpotential (337 mV) than the Ni_10_Co-BTC/KB-derived electrocatalyst (347 mV). This illustrates that the conductivity, which could have been increased by introducing KB, is not the key factor limiting the OER activity of the Ni_10_Co-BTC-derived electrocatalyst. However, a clearly positive effect of KB in the Ni_10_Co-BTC/KB-derived material is a decreased Tafel slope with 70 mV dec^−1^ in comparison to the Ni_10_Co-BTC-derived material with 87 mV dec^−1^, which indicates a more favorable kinetics of the OER for the composite-derived material. The Ni_10_Fe-BTC-dervied electrocatalyst remains the most stable material in the electrochemical OER performance (η = 346 mV → 344 mV) and has the lowest Tafel slope of 47 mV dec^−1^, showing that the activity of Ni-electrocatalysts can be improved to some extent with incorporated iron. The results of the Tafel analysis show that the introduction of KB in the Ni_10_Co-BTC MOF facilitates to overcome the kinetic barrier of the complex four electron-proton coupled OER transfer process. The composite material Ni_10_Co-BTC/KB and the presented protocol give insight into the possibility of combining MOFs, as sacrificial agents, with KB to generate new MOF-based electrocatalysts for electrocatalytic reactions. Further research should now be conducted to investigate potential other nickel-metal combinations to optimize the electrocatalytic performance.

## Figures and Tables

**Table 1:** SEM-EDX and AAS results of the mixed-metal samples.

Sample	SEM-EDX		AAS *			
	Molar Ratio		Metal wt.%		Approximate Molar Ratio	
	Ni	Fe/Co	Ni	Fe/Co	Ni	Fe/Co
Ni_10_Fe-BTC	9	1	16.7	1.5	11	1
Ni_10_Co-BTC	7	1	16.3	1.5	11	1
Ni_10_Co-BTC/KB	7	1	10.6	1.3	8	1

* Atomic absorption spectroscopy. Weighted samples were heated to dryness with aqua regia for two times and afterwards stirred with aqua regia overnight. The solution was carefully filtered and diluted with Millipore water to a volume of 25 mL. The resulting solutions were further diluted with Millipore water (1:50) for the AAS measurements.

## Data Availability

The data presented in this study are available on request from the corresponding author.

## Supplementary Materials

The following supporting information can be downloaded online. Table S1. Elemental analysis of the MOF samples. Table S2. Assignments of IR-bands of Ni-BTC analogs (cm^−1^). Table S3. BET-surface areas and total pore volumes of the Ni-BTC analogs. Table S4. Overpotentials (at 10 mA/cm^2^) and Tafel slopes for Ni_10_Fe-BTC, Ni_10_Co-BTC, Ni_10_Co-BTC/KB, KB and Ni/NiO nanoparticles done with a GCE (loading: 0.2 mg/cm^2^) with a scan rate of 5 mV/s in a 1.0 mol/L KOH electrolyte. Figure S1. TGA curves of (a) Ni_10_Fe-BTC, (b) Ni_10_Co-BTC, (c) Ni_10_Co-BTC/KB and (d) KB under N_2_ atmosphere with a heating rate of 5 K/min. Figure S2. SEM-EDX spectra of (a) Ni_10_Fe-BTC, (b) Ni_10_Co-BTC and (c) Ni_10_Co-BTC/KB. Figure S3. (a,b) SEM images and (c) SEM-EDX spectra of KB. Figure S4. TEM images of Ni_10_Fe-BTC (a) before (shown particle size: 3.1 μm) and (b–d) after 1000 CVs. Figure S5. TEM images of Ni_10_Co-BTC (a) before and (b–d) after 1000 CVs (shown particle size in (b): 2.8 μm; displayed particles in (c) give the average diameter of 20 nm ± 9 nm; (d) the lattice spacings and grain boundaries are illustrated in red and red lines, respectively). Figure S6. TEM images of Ni_10_Co-BTC/KB (a) before (shown particle size: 4.4 μm) and (b,c) after 1000 CVs ((c) the lattice spacings and grain boundaries are illustrated in red and red lines, respectively.) and (d) histogram of Ni_10_Co-BTC/KB after 1000 CVs determined from (b) give the average diameter of 5 nm ± 1 nm (1σ). Scheme S1. Schematic relation between β -Ni(OH)_2_, α-Ni(OH)_2_, β-NiOOH and γ-NiOOH. Figure S7. FT-IR spectra of (a) Ni_10_Co-BTC after 24 h in 1 mol/L KOH (dark green) and (b) comparison with Ni_10_Co-BTC (green), (c) Ni_10_Co-BTC/KB after 24 h in 1 mol/L KOH (brown) and (d) comparison with Ni_10_Co-BTC/KB (orange), (e) Ni_10_Fe-BTC after 24 h in 1 mol/L KOH (purple) and (f) comparison with Ni_10_Fe-BTC (red). Table S5. Assignments of IR-bands of Ni-BTC analogs after 24 h in 1 mol/L KOH (cm^−1^). References [82,83,84] are cited in the supplementary materials.
