# Supplementary material for: Nickel-Based Metal-Organic Frameworks as Electrocatalysts for the Oxygen Evolution Reaction (OER)
Source: Molecules. 2022 Feb 12;27(4):1241. doi: 10.3390/molecules27041241 (PMC8875730; doi:10.3390/molecules27041241)
Supplement: Supplementary file 1 [file molecules-27-01241-s001.zip › molecules-1586504-supplementary.pdf]

Supporting Information

for

Nickel-based metal-organic frameworks as electro-catalysts for the oxygen evolution reaction (OER)

Linda Sondermann [1], Wulv Jiang [2], Meital Shviro [2,*], Alex Spieß [1], Dennis Woschko [1], Lars Rademacher [1] and Christoph Janiak [1,*]

[1] Institut für Anorganische Chemie und Strukturchemie, Heinrich-Heine-Universität Düsseldorf, 40225 Düsseldorf, Germany; linda.sondermann@hhu.de (L.S.); alex.spiess@hhu.de (A.S.); dennis.woschko@hhu.de (D.W.); lars.rademacher@hhu.de (L.R.)

[2] Forschungszentrum Jülich GmbH, Institute of Energy and Climate Research, IEK-14: Electrochemical Process Engineering, 52425 Jülich, Germany; j.wulv@fz-juelich.de

* Correspondence: m.shviro@fz-juelich.de (M.S.); janiak@uni-duesseldorf.de (C.J.); Tel.: +49-211-81-12286 (C.J.)

Additional experimental data

Table S1. Elemental analysis of the MOF samples.[a]

Sample	C [%]	H [%]	N [%]
**Ni_{10}Fe-BTC**	38.43	3.59	5.35
**Ni_{10}Co-BTC**	38.51	3.80	7.13
**Ni_{10}Co-BTC/KB**	51.13	3.04	5.25
**Ni-BTC = [$Ni_3(BTC)_2$] calculated**	36.63	1.02	--
**[$Ni_3(BTC)_2(Me_2NH)_3$] calculated [b]**	39.72	3.75	5.79

[a] Samples have been dried at 120 °C for 12 h under vacuum before analysis. [b] Our syntheses of Ni_{10}Fe- and Ni_{10}Co-BTC followed the synthesis of Ni-BTC in ref. [1], which was obtained as the dimethylamine adduct with Me_2NH being a decomposition/hydrolysis product of DMF. In general, CHN elemental analyses of MOFs deviate from the framework formula due to unavoidable residual solvent molecules or re-adsorbed moisture during storage and handling.

Table S2. Assignments of IR-bands of Ni-BTC analogs (cm^{-1}).

Allocation*	$Ni_{10}Fe$-BTC	$Ni_{10}Co$-BTC	$Ni_{10}Co$-BTC/KB
ν(OH) (of for example water) [1,2]	3377	3398	3410
ν(C-H) [1,2,3]	2971	2967	3003
	–	2930	2923
ν(N-H) [2,4]	2803	2804	2803
	2486	2492	–
ν(C=O) (of DMF) [5] / δ(O-H) [6]	–	1654	1650
ν_{as}(OCO) [4,7]	1617	–	–
	1556	1563	1562
ν_{s}(OCO) [4,7]	1435	1438	1439
	1364	1369	1370
ν(C-N) (of DMF) [8]	–	1253	1252
ν(C-N) (of DMF)[5,9] / $\nu(C\text{-}C)_{Ar}$ [4]	1102	1103	1098
$\varrho(C\text{-}H)_{Ar}$ [8]	1046	–	1064
	1023	1025	1026
ν(CN-CHO) (of DMF) [5,10]	932	935	937
	904	908	908
$\delta(C\text{-}H)_{Ar}$ [1,4,8] / $\nu(C\text{-}C)_{Ar}$ [4]	882	–	–
	768	766	764
	718	720	721
ν(Fe_2Ni-O) [11], ν(Co-O) [12]	718	720	721
	–	665	666
ν(Ni-O) [6]	573	577	–
	461	466	467

* ν = stretching vibration (ν_{as}= asymmetric, ν_s = symmetric vibration); δ = bending vibration (ϱ = in plane, γ = out of plane vibration); $_{Ar}$ = aromatic vibration

Table S3. BET-surface areas and total pore volumes of the Ni-BTC analogs.

	$Ni_{10}Fe$-BTC	$Ni_{10}Co$-BTC	$Ni_{10}Co$-BTC/KB	KB
BET-surface area (m^2/g)	555	303	596	1415
Total pore volume (cm^3/g)*	0.24	0.15	0.45	1.59

*The total pore volumes were determined at p/p_0 = 0.90 from the adsorption branch for pores ≤ 23 nm.

Table S4. Overpotentials (at 10 mA/cm^2) and Tafel slopes for Ni_{10}Fe-BTC, Ni_{10}Co-BTC, Ni_{10}Co-BTC/KB, KB and Ni/NiO nanoparticles done with a GCE (loading: 0.2 mg/cm^2) with a scan rate of 5 mV/s in a 1.0 mol/L KOH electrolyte.

**electrocatalyst**	**η (at j = 10 mA/cm^2), vs. RHE) (mV)**		**Tafel slope (mV dec^{-1})**
	before 1000 cycles	after 1000 cycles	
Ni_{10}Fe-BTC	346	344	47
Ni_{10}Co-BTC	378	337	87
Ni_{10}Co-BTC/KB	366	347	70
KB	376	422	93
Ni/NiO	370	358	67

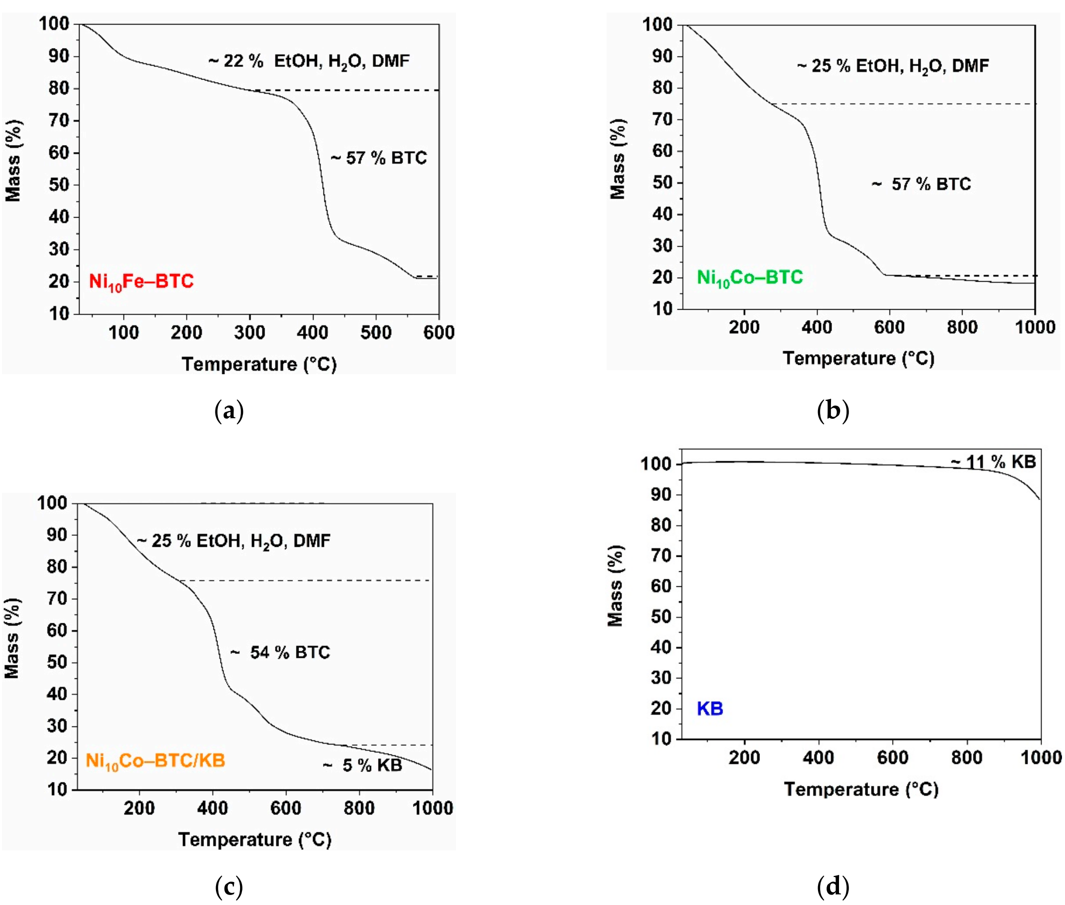

Figure S1. TGA curves of (**a**) Ni_{10}Fe-BTC, (**b**) Ni_{10}Co-BTC, (**c**) Ni_{10}Co-BTC/KB and (**d**) KB under N_2 atmosphere with a heating rate of 5 K/min.

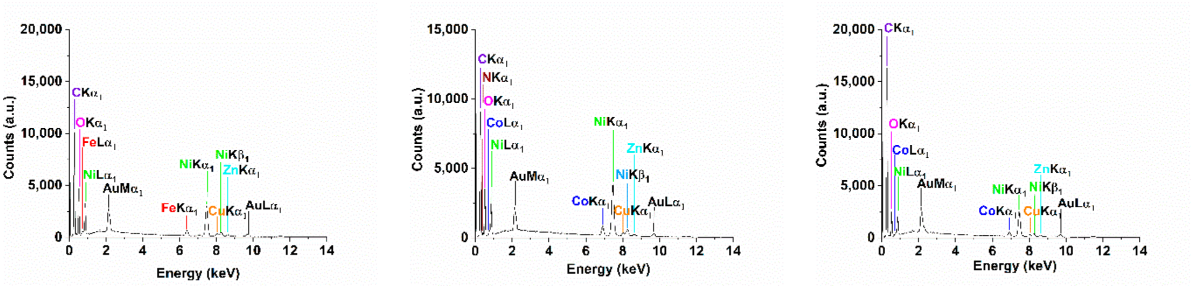

(**a**) (**b**) (**c**)

Figure S2. SEM-EDX spectra of (**a**) $Ni_{10}Fe$-BTC, (**b**) $Ni_{10}Co$-BTC and (c) $Ni_{10}Co$-BTC/KB.

The small amount of gold, copper and zinc found in the SEM-EDX spectra (Figure S2, Figure S3) is due to the brass sample holder and the sputtering of the sample with gold prior to the investigation. Nitrogen can be traced to 2-MeImH and/or to incorporated DMF solvent molecules. It has been reported that solvent molecules such as DMF can occupy the axial positions of the metal centers in HKUST-1 based structures [1,3].

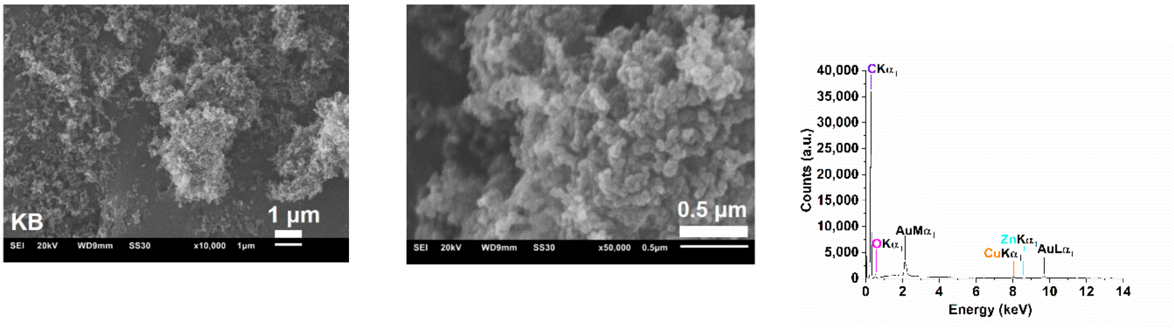

(a) (b) (c)

Figure S3. (**a**,**b**) SEM images and (**c**) SEM-EDX spectra of KB.

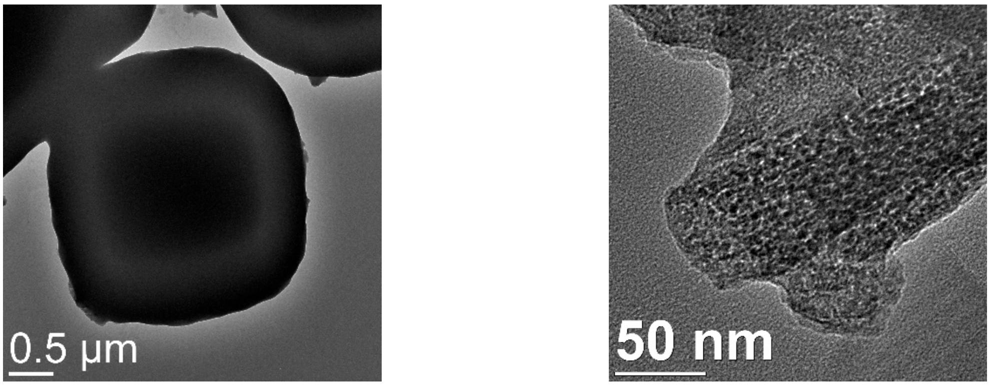

(**a**) (**b**)

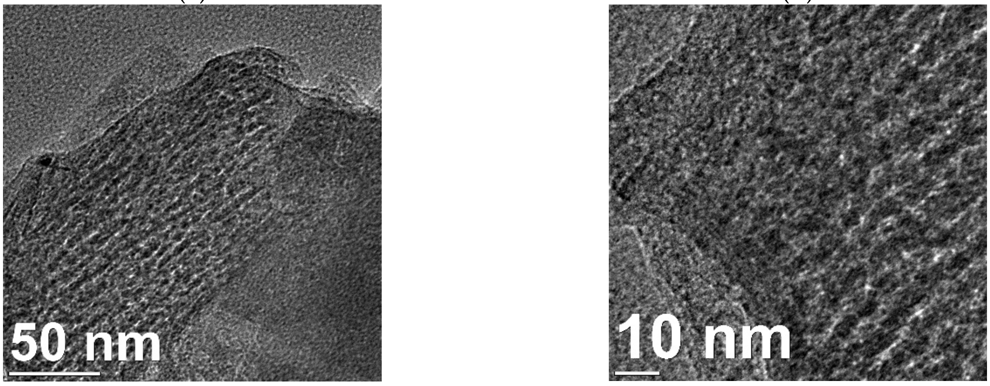

(**c**) (**d**)

Figure S4. TEM images of $Ni_{10}Fe$-BTC (**a**) before (shown particle size: 3.1 μm) and (**b**–**d**) after 1000 CVs.

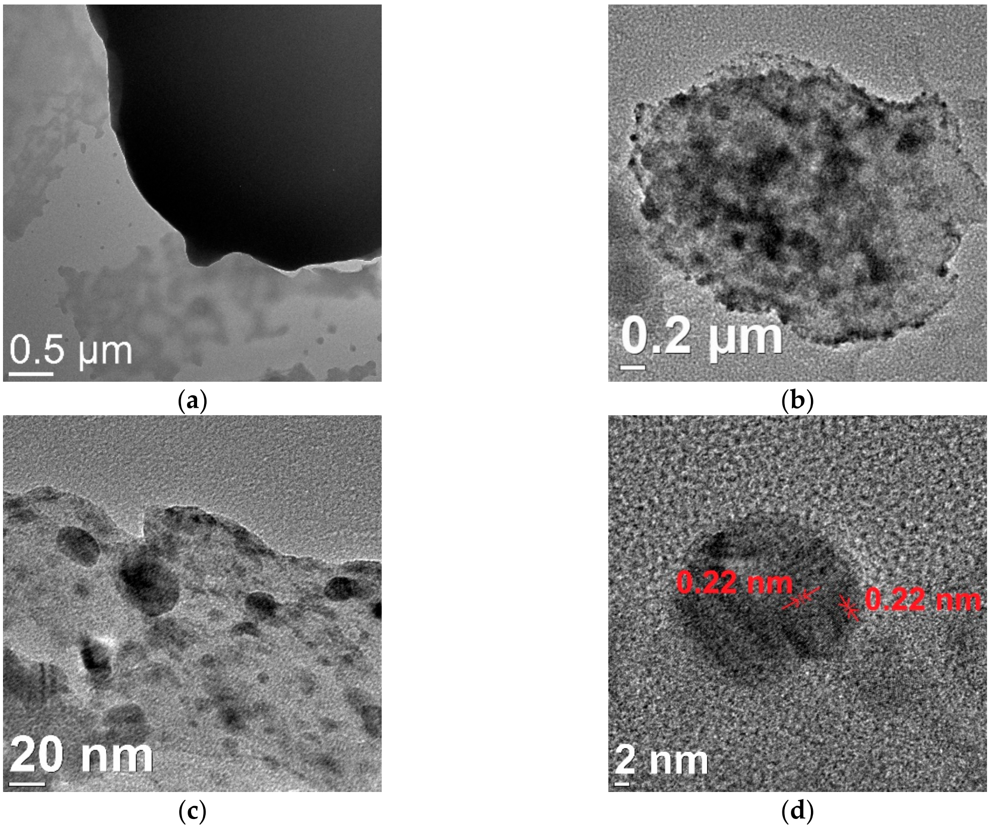

Figure S5. TEM images of $Ni_{10}Co$-BTC (**a**) before and (b-d) after 1000 CVs (shown particle size in (**b**): 2.8 µm; displayed particles in (**c**) give the average diameter of 20 nm ± 9 nm; (**d**) the lattice spacings and grain boundaries are illustrated in red and red lines, respectively).

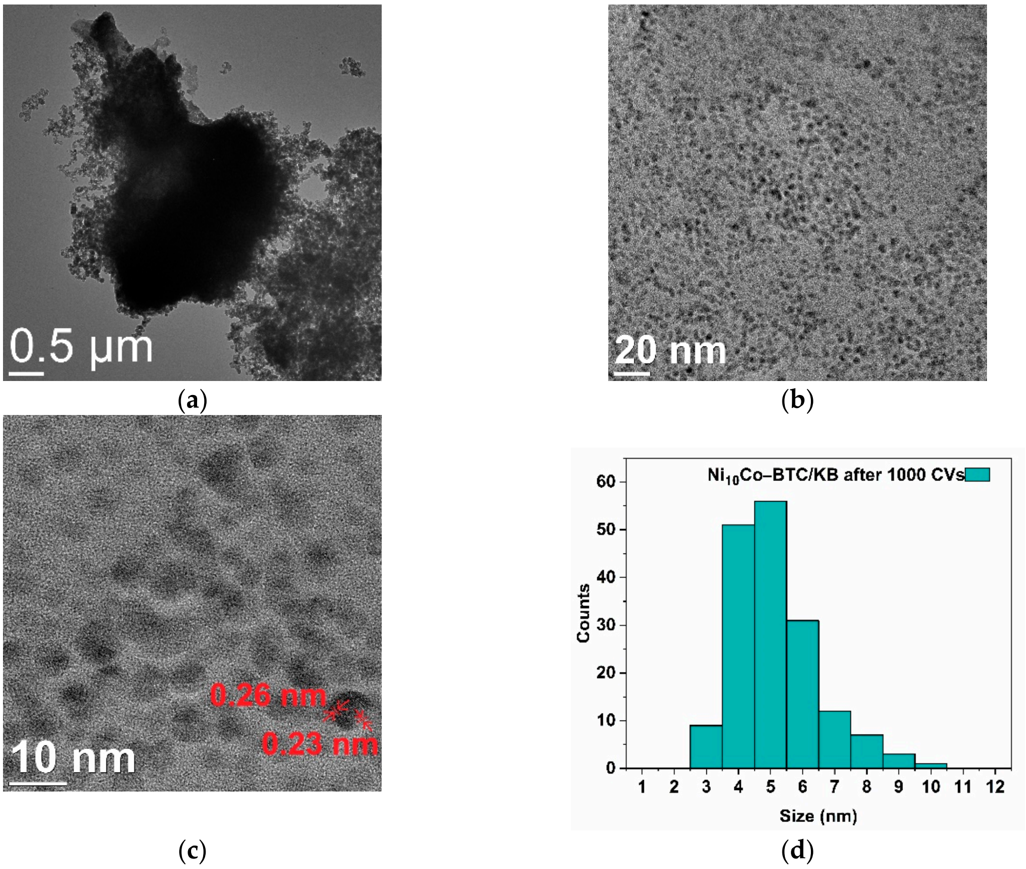

Figure S6. TEM images of $Ni_{10}Co$-BTC/KB (**a**) before (shown particle size: 4.4 µm) and (**b**,**c**) after 1000 CVs ((**c**) the lattice spacings and grain boundaries are illustrated in red and red lines, respectively.) and (**d**) histogram of $Ni_{10}Co$-BTC/KB after 1000 CVs determined from (**b**) give the average diameter of 5 nm ± 1 nm (1σ).

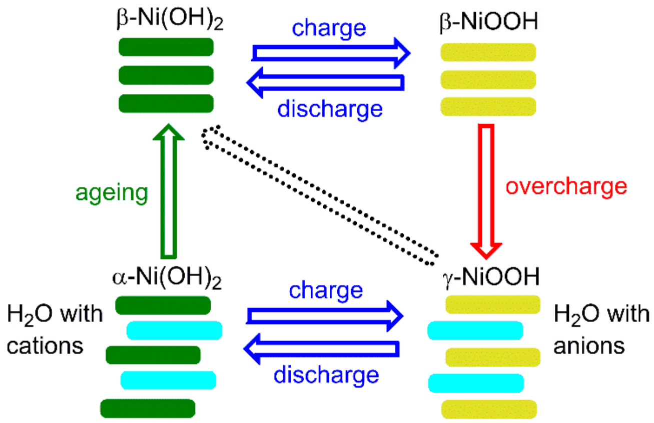

Scheme S1. Schematic relation between β -Ni(OH)₂, α-Ni(OH)₂, β-NiOOH and γ-NiOOH.

α-$Ni(OH)_2$ is unstable and can transform into the thermodynamically favored β-$Ni(OH)_2$ over time (chemical ageing process) [13]. The difference between α- and β-$Ni(OH)_2$ is that α-$Ni(OH)_2$ consists of β-$Ni(OH)_2$ layers, intercalated by water (and possibly additional other) molecules and has a turbostratic structure [13,14]. In an electrochemical process α/β-$Ni(OH)_2$ can be converted into γ/β-NiOOH, while β-NiOOH also can change into γ-NiOOH [13,14]. Considering that the oxygen evolution reaction (OER) takes place while charging in an electrochemical process, would strengthen the assumption, that β-NiOOH and γ-NiOOH are also likely present inside the MOF-derived materials after the electrochemical measurements, which could also be seen in the XRD diffraction patterns of the samples after soaking for 24 h in 1 mol/L KOH (Figure 6) [15].

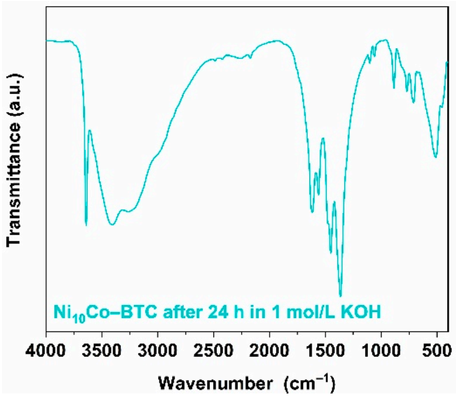

(a)

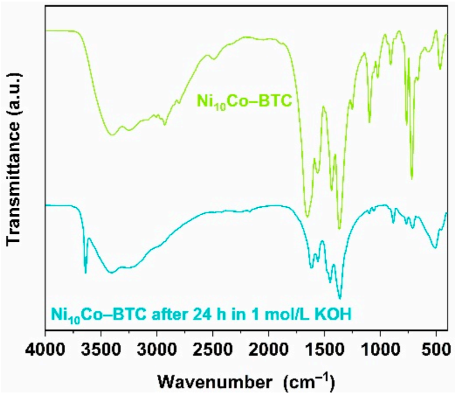

(b)

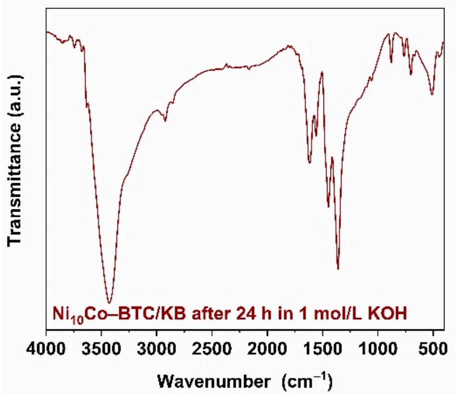

(c)

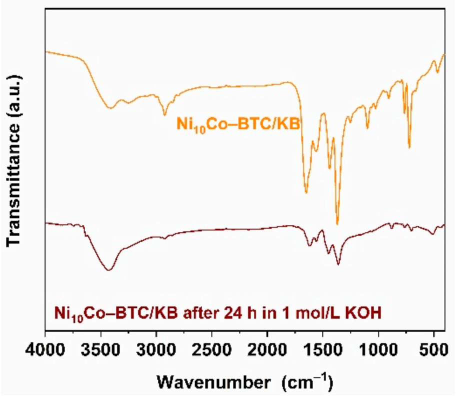

(d)

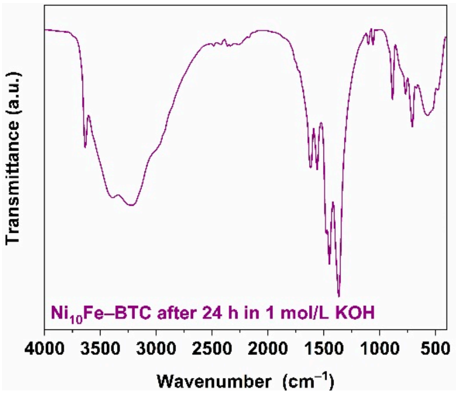

(e)

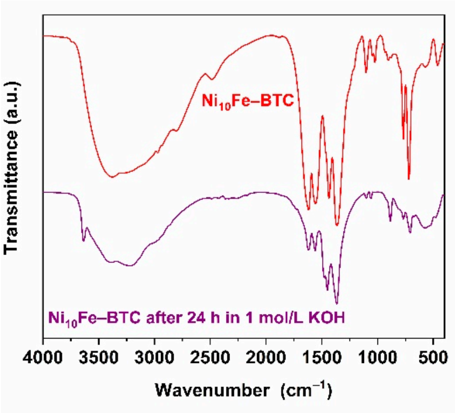

(f)

Figure S7. FT-IR spectra of (**a**) $Ni_{10}Co$-BTC after 24 h in 1 mol/L KOH (dark green) and (**b**) comparison with $Ni_{10}Co$-BTC (green), (**c**) $Ni_{10}Co$-BTC/KB after 24 h in 1 mol/L KOH (brown) and (**d**) comparison with $Ni_{10}Co$-BTC/KB (orange), (**e**) $Ni_{10}Fe$-BTC after 24 h in 1 mol/L KOH (purple) and (**f**) comparison with $Ni_{10}Fe$-BTC (red).

Table S5. Assignments of IR-bands of Ni-BTC analogs after 24 h in 1 mol/L KOH (cm^{-1}).[a]

Allocation*[13]		$Ni_{10}Fe$-BTC after 24 h in 1 mol/L KOH	$Ni_{10}Co$-BTC after 24 h in 1 mol/L KOH	$Ni_{10}Co$-BTC/KB after 24 h in 1 mol/L KOH
α-$Ni(OH)_2$	β-$Ni(OH)_2$			
–	–	–	–	3845
–	–	–	–	3743
–	A_{2u} (LO) O–H stretch	–	–	3677
O–H stretch, lattice OH/layer H_2O	A_{2u} (TO) O–H stretch / disordered (TO) O–H stretch	3637	3639	3631
O–H stretch, free H_2O	O–H stretch	3391	3403	3429
O–H stretch, free H_2O	–	3215	3262	2955
–	–	2487	2486	2923
–	–	2421	2421	2856
–	–	2363	–	2351
–	–	2336	–	–
–	–	2266	2262	–
–	–	2172	2170	2167
–	–	–	–	1738
O–H bend, free H_2O	O–H bend, free H_2O	1618	1616	1616
–	–	1560	1560	1559
O–H bend, lattice OH	–	1478	–	–
–	–	1450	1450	1448
O–H bend, lattice OH	–	1366	1364	1361
–	–	1101	1102	1105
combination lattice mode	E_u (LO) O–H bend	1060	1059	1057
combination lattice mode	–	885	885	880
–	–	768	769	763
–	–	708	709	702
lattice mode	E_u (LO) O–H bend	672	–	670
–	E_u (TO) O–H bend	570	512	510
lattice mode	E_u (LO)/A_{2u} (LO) lattice modes	480	458	446

[a] All major IR bands are listed for the decomposition product of $Ni_{10}Fe$/Co-BTC after 24 in 1 mol/L KOH. The bands which are presumable derive from α- and β-$Ni(OH)_2$ were assigned. * LO = longitudinal optical component; TO = transverse optical component; $_u$= odd vibrational mode (u for the German "ungerade")

References

1. Maniam, P.; Stock, N. Investigation of Porous Ni-Based Metal-Organic Frameworks Containing Paddle-Wheel Type Inorganic Building Units via High-Throughput Methods. *Inorg. Chem.* **2011**, *50*, 5085-5097. DOI:10.1021/ic200381f
2. Meier, H.; Bienz, S.; Bigler, L.; Fox, T. *Spektroskopische Methoden in der organischen Chemie*, 8 ed., Georg Thieme, Stuttgart, Germany, **2011**.

3. Wade, C. R.; Dincă, M. Investigation of the synthesis, activation, and isosteric heats of CO_2 adsorption of the isostructural series of metal–organic frameworks $M_3(BTC)_2$ (M = Cr, Fe, Ni, Cu, Mo, Ru). *Dalton Trans.* **2012**, *41*, 7931-7938. DOI:10.1039/c2dt30372h
4. Wu, Y.; Song, X.; Li, S.; Zhang, J.; Yang, X.; Shen, P.; Gao, L.; Wei, R.; Zhang, J.; Xiao, G. 3D-monoclinic M–BTC MOF (M = Mn, Co, Ni) as highly efficient catalysts for chemical fixation of CO_2 into cyclic carbonates. *J. Ind. Eng. Chem.* **2018**, *58*, 296-303. DOI:10.1016/j.jiec.2017.09.040
5. Israr, F.; Kim, D. K., Kim; Y., Chun, W. Scope of various solvents and their effects on solvothermal synthesis of Ni-BTC. *Quim. Nova* **2016**, *39*, 669-675. DOI:10.5935/0100-4042.20160068
6. Maruthapandian, V.; Kumaraguru, S.; Mohan, S.; Saraswathy, V.; Muralidharan, S. An Insight on the Electrocatalytic Mechanistic Study of Pristine Ni MOF (BTC) in Alkaline Medium for Enhanced OER and UOR. *ChemElectroChem* **2018**, *5*, 2795-2807. DOI:10.1002/celc.201800802
7. Yaghi, O. M.; Li, H.; Groy, T. L. Construction of Porous Solids from Hydrogen-Bonded Metal Complexes of 1,3,5-Benzenetricarboxylic Acid. *J. Am. Chem. Soc.* **1996**, *118*, 9096-9101. DOI:10.1021/ja960746q
8. Pretsch, E.; Bühlmann, P.; Badertscher, M. *Spektroskopische Daten Zur Strukturaufklärung organischer Verbindungen,* 5th ed.; Springer: Berlin/Heidelberg, Germany, **2010**.
9. Gan, Q.; He, H.; Zhao, K.; He, Z.; Liu, S. Morphology-dependent electrochemical performance of Ni-1,3,5-benzenetricarboxylate metal-organic frameworks as an anode material for Li-ion batteries. *J. Colloid Interface Sci.* **2018**, *530*, 127-136. DOI:10.1016/j.jcis.2018.06.057
10. Israr, F.; Chun, D.; Kim, Y.; Kim, D. K. High yield synthesis of Ni-BTC metal–organic framework with ultrasonic irradiation: Role of polar aprotic DMF solvent. *Ultrason. Sonochem.* **2016**, *31*, 93-101. DOI:10.1016/j.ultsonch.2015.12.007
11. Vuong, G.-T.; Pham, M.-H.; Do, T.-O. Direct synthesis and mechanism of the formation of mixed metal Fe_2Ni-MIL-88B. *CrystEngComm* **2013**, *15*, 9694-9703. DOI:10.1039/c3ce41453a
12. He, J.; Lu, X.; Yu, J.; Wang, L.; Song, Y. Hierarchical $Co(OH)_2$ nanostructures/glassy carbon electrode derived from Co(BTC) metal–organic frameworks for glucose sensing. *J. Nanopart. Res.* **2016**, *18*, 184. DOI:10.1007/s11051-016-3489-8
13. Hall, D. S.; Lockwood, D. J.; Bock, C.; MacDougall, B. R. Nickel hydroxides and related materials: a review of their structures, synthesis and properties. *Proc. R. Soc. A* **2015**, *471*, 20140792. DOI:10.1098/rspa.2014.0792
14. Young, K.-H.; Wang, L.; Yan, S.; Liao, X.; Meng, T.; Shen, H.; Mays, W.C. Fabrications of High-Capacity Alpha-$Ni(OH)_2$. *Batteries* **2017**, *3*, 6. DOI:10.3390/batteries3010006
15. Lu, C.-T.; Chiu, Y.-W.; Li, M.-J.; Hsueh, K.-L.; Hung, J.-S. Reduction of the Electrode Overpotential of the Oxygen Evolution Reaction by Electrode Surface Modification. *Int. J. Electrochem.* **2017**, *2017*, 7494571. DOI:10.1155/2017/7494571
